# LINKS BETWEEN SOCIAL ENGAGEMENT AND SUCCESSFUL AGING AMONG AFFORDABLE SENIOR HOUSING RESIDENTS

**DOI:** 10.1093/geroni/igz038.2304

**Published:** 2019-11-08

**Authors:** Noah J Webster, Noah Webster, Toni Antonucci

**Affiliations:** University of Michigan, Ann Arbor, Michigan, United States

## Abstract

We report on a study designed to integrate the convoy model of social relations with the successful aging model. The combined approach draws on the natural resources of interpersonal relations to promote successful aging thus minimizing risk of disease and disability, enhancing\maintaining mental and physical functioning, and facilitating continued engagement in life. We describe the MacHouse Affordable Housing Study, a field experiment conducted in an affordable senior housing community. The experiment involved a resident driven intensive intervention program designed to increase social activities and engagement. Based on an extensive needs assessment and interviews with the resident council as well as individual residents a program of activities was developed. This included group meals, an exercise program that included a walking group and an exercise class, communal gardens, computer classes and resident outings. Results indicate that more frequent participation in these activities was associated with increased life satisfaction over time.

